# Procalcitonin and CRP as Predictors of Anastomotic Dehiscence in Adult Patients

**DOI:** 10.7759/cureus.88190

**Published:** 2025-07-17

**Authors:** Harvey Y Zamora Veliz, Rebeca Y Paredes Cabanillas, Javier E Gomez Trejo, Victor G Chavarin Covarrubias

**Affiliations:** 1 General Surgery, Institute for Social Security and Services for State Workers (ISSSTE) “Mazatlán Clinic Hospital”, Mazatlán, MEX

**Keywords:** anastomotic leak, c-reactive protein, dehiscence, early markers, procalcitonin

## Abstract

Introduction

Intestinal anastomotic leakage is a serious postoperative complication associated with high morbidity and mortality. Although acute-phase reactants such as procalcitonin (PCT) and CRP have been proposed as early biomarkers, their predictive utility remains insufficiently established in low-resource settings. This study aimed to evaluate the usefulness of PCT and CRP in detecting anastomotic leakage in adult patients undergoing intestinal surgery at a secondary-level hospital.

Methods

A retrospective, cross-sectional, and analytical case series was conducted based on the medical records of adult patients who underwent intestinal anastomosis between July 2022 and July 2024 at a secondary-level public hospital in Mexico. Thirty patients met the inclusion criteria. Serum levels of PCT and CRP were measured on postoperative days 1, 3, and 5. Descriptive and inferential statistical analyses were performed. The Holm-Bonferroni method was applied to adjust for multiple comparisons. A p-value < 0.05 was considered statistically significant.

Results

Five patients (16.7%) developed anastomotic leakage. On postoperative day five, mean PCT levels were significantly higher in patients with leakage (9.02 ± 15.92 ng/mL) compared to those without leakage (2.63 ± 1.52 ng/mL; unadjusted p = 0.044), although this difference lost significance after adjustment for multiple comparisons. CRP levels followed a similar upward trend in the leakage group (288.45 ± 223.67 mg/L vs. 186.41 ± 74.83 mg/L; p = 0.067), but the difference was not statistically significant. Leakage occurred more frequently in colorectal anastomoses and was associated with longer hospital stays, higher reoperation rates, and increased mortality, though none of these associations reached statistical significance.

Conclusion

Serial measurement of PCT, and to a lesser extent, CRP, may support the early detection of anastomotic leakage after intestinal surgery. However, given the small sample size and loss of statistical significance after correction, these findings should be interpreted with caution. Larger prospective studies are needed to validate these preliminary observations and determine their clinical applicability in broader surgical settings.

## Introduction

Anastomotic leakage remains one of the most feared complications following intestinal surgery, as it is associated with increased morbidity, prolonged hospitalization, higher reoperation rates, and a mortality rate ranging from 1.6% to 12% [1,2].

Despite advances in surgical techniques and perioperative care, early identification of this complication continues to be challenging, particularly in secondary-level hospitals with limited access to advanced imaging and diagnostics [3,4].

Although clinical signs such as fever, abdominal pain, tachycardia, or abnormal drainage output may raise suspicion of an anastomotic leak, these manifestations often occur late in the course of the complication, potentially delaying treatment and adversely affecting outcomes [5-7]. In this context, identifying reliable and accessible biomarkers for early detection is essential to improving postoperative management.

Among the most studied inflammatory markers are CRP and procalcitonin (PCT). Under normal physiological conditions, CRP levels remain below 10 mg/L and PCT levels are <0.05 ng/mL. Both increase significantly in response to infection or systemic inflammation [8,9]. CRP has been widely used due to its high sensitivity and availability, though its specificity in identifying severe complications such as anastomotic dehiscence remains variable [9,10]. In contrast, PCT has shown greater specificity for bacterial infections and sepsis, making it a promising marker for early postoperative complications, including anastomotic leakage [8,9].

Recent studies have explored the diagnostic performance of serial measurements of CRP and PCT, suggesting that their trends may help predict the onset of leakage before clinical deterioration becomes evident [8,10]. However, most of these studies are from high-resource settings. Evidence remains scarce regarding their applicability in secondary-level hospitals, where access to confirmatory imaging is limited and clinical decision-making often relies on basic laboratory findings.

Therefore, the aim of this study is to assess the utility of PCT and CRP as early predictors of anastomotic leakage in adult patients undergoing intestinal surgery in a secondary-level hospital. We hypothesize that serial measurements of these biomarkers may provide a practical and cost-effective method to support early diagnosis and timely intervention in resource-constrained settings.

## Materials and methods

Study design and setting

This retrospective, cross-sectional, analytical, and observational case series was based on data extracted from the electronic medical records of patients who underwent intestinal anastomosis procedures performed by the Departments of General Surgery, Coloproctology, and Oncosurgery at the General Hospital “Dr. Aquiles Calles Ramírez” in Tepic, Nayarit, Mexico, between July 2022 and July 2024.

Sample selection

A total of 30 adult patients were included. A non-probabilistic convenience sampling method was employed. Eligible cases involved patients who underwent intestinal surgery with anastomosis, regardless of whether they developed anastomotic dehiscence.

The unit of analysis was each patient who met the inclusion criteria and underwent an intestinal anastomosis during the study period. The unit of observation was the corresponding medical record.

Inclusion Criteria

Patients included in the study were aged 18 years or older, of any sex. Eligible participants had undergone elective or emergency intestinal resection with either manual or mechanical anastomosis performed by the designated surgical departments. Patients with pre-existing stomas who underwent intestinal reconnection were also included. Additionally, inclusion required the availability of postoperative serum PCT and CRP levels on postoperative days 1, 3, and 5.

Exclusion Criteria

Medical records lacking complete postoperative PCT and CRP data.

Elimination Criteria

Illegible or incomplete medical records were excluded from the study.

Of the 174 records initially identified, 144 were excluded for not meeting the inclusion criteria. Specifically, 62 patients lacked serial measurements of CRP and PCT on postoperative days 1, 3, and 5; 39 had undergone procedures that did not involve intestinal anastomosis; 22 were under 18 years of age; 11 had incomplete or illegible records; and 10 had undergone surgeries outside the Departments of General Surgery, Coloproctology, or Oncosurgery. After this screening process, 30 patients were deemed eligible and included in the final analysis.

Variables collected

The variables collected included demographic information such as age, sex, and comorbidities. Surgical details were also recorded, including the department performing the surgery, type of surgery, anastomosis technique, and anatomical location. Laboratory data consisted of serial measurements of CRP and PCT levels on postoperative days 1, 3, and 5. Outcome variables included the presence of anastomotic dehiscence, postoperative complications, length of hospital stay, and mortality.

Reference values were considered normal for PCT at <0.05 ng/mL and for CRP at <10 mg/L.

Data were entered into a custom data collection sheet and compiled using Microsoft Excel (version 16.16.27, 201012). The final dataset was then imported into IBM SPSS Statistics version 26 for statistical analysis.

Statistical analysis

Descriptive statistics were calculated for all variables. Categorical variables were expressed as frequencies and percentages. Continuous variables were assessed for normality using the Shapiro-Wilk test. Parametric variables were reported as mean ± SD, and non-parametric variables as median and IQR.

Comparative analyses were performed using Student’s t-test for continuous variables (PCT and CRP levels) between patients with and without anastomotic leakage. The chi-square test or Fisher’s exact test, as appropriate, was used to examine associations between categorical variables and mortality.

Given the multiple comparisons across different postoperative days (1, 3, and 5), p-values were adjusted using the Holm-Bonferroni correction method to control for the family-wise error rate. A corrected p-value < 0.05 was considered statistically significant.

Ethical considerations

This study was approved by the Institutional Research Committee of the General Hospital “Dr. Aquiles Calles Ramírez” on November 12, 2024. Since the research involved the analysis of anonymized, pre-existing medical records, it was classified as risk-free in accordance with Article 17 of the Mexican General Health Law for Health Research.

All patient data were handled in compliance with the Mexican Federal Law on Protection of Personal Data and the NOM-004-SSA3-2012 regulation for clinical records (Sections 5.4, 5.5, and 5.7). Personal identifiers were removed, and no information was disclosed to third parties.

## Results

A total of 30 patients were included in this study, with a mean age of 67.20 ± 12.54 years (range: 45-90). The majority were male (63.3%). The most common comorbidities were cardiovascular diseases (40%), followed by endocrine, genitourinary, and pulmonary conditions. A portion of patients (36.7%) had no documented comorbidities (Table 1).

**Table 1:** Age, sex, and comorbidities of patients undergoing intestinal anastomosis (n = 30).

Variable	Overall Values	Chi-square (p-value)
Age (years), mean ± SD	67.2 ± 12.54	-
Male sex, n (%)	19 (63.3%)	-
Female sex, n (%)	11 (36.7%)	-
Comorbidities, n (%)		
Endocrine	5 (16.7%)	0.387
Cardiovascular	12 (40.0%)	0.309
Genitourinary	5 (16.7%)	0.372
Pulmonary	4 (13.3%)	0.435
Neurological	1 (3.3%)	0.567
Autoimmune	1 (3.3%)	0.276

The most common referring service overall was oncologic surgery (40%), followed by general surgery (36.6%) and proctology (23.3%).

Colon or sigmoid tumors represented the most common surgical indication (36.6%), followed by intestinal ischemia (20%) and diverticular disease (13.3%). Intestinal reconnection procedures accounted for 30% of cases.

Most procedures were elective (70%), and mechanical anastomosis was the predominant technique (80%). Colo-rectal anastomosis was the most frequent site (33.3%), followed by jejuno-ileal and ileo-ileal locations (20%).

The most frequently used anastomotic configuration was end-to-end (60%), followed by side-to-side (26.6%). There were no statistically significant differences in configuration type between patients with and without anastomotic leakage (p = 0.918) (Table 2).

**Table 2:** Patient origin service, diagnosis, type of surgery performed, type of anastomosis, level of anastomosis, and class of anastomosis (n = 30).

Characteristics	Total (n = 30)	Chi-square (p-value)
Origin service		
General surgery	11 (36.7%)	-
Oncosurgery	12 (40.0%)	0.185
Proctology	7 (23.3%)	-
Patient diagnosis		
Intestinal ischemia	6 (20.0%)	0.265
Colon or sigmoid tumors	11 (36.7%)	0.289
Diverticular disease	4 (13.3%)	0.508
Intestinal reconnection	9 (30.0%)	0.313
Type of surgery		
Elective	21 (70.0%)	0.291
Emergency	9 (30.0%)	-
Type of anastomosis		
Manual	6 (20.0%)	-
Mechanical	24 (80.0%)	0.313
Anastomosis level		
Colo-colonic	2 (6.7%)	-
Colo-rectal	10 (33.3%)	0.64
Esophago-jejunal	1 (3.3%)	-
Ileo-ileal	6 (20.0%)	-
Ileo-colic	4 (13.3%)	-
Ileo-rectal	1 (3.3%)	-
Jejuno-ileal	6 (20.0%)	-
Anastomosis class		
Side-to-side	8 (26.7%)	-
Side-to-end	1 (3.3%)	-
End-to-side	3 (10.0%)	-
End-to-end	18 (60.0%)	0.104

The overall rate of anastomotic leakage was 16.7%. Other postoperative complications included seroma (3.3%), sepsis or intra-abdominal abscess (13.3%), and eventration (3.3%), with no statistically significant associations between these events and anastomotic leakage (Table 3).

**Table 3:** Frequency of dehiscence and other anastomosis complications (n = 30).

Complication	Total (n = 30)	P-value	Chi-square
Frequency of dehiscence			
Yes	5 (16.7%)	0.628	0.273
No	25 (83.3%)	-	-
Other complications			
Seroma	1 (3.3%)	1	0.567
Sepsis or abscess	4 (13.3%)	0.29	0.177
Eventration	1 (3.3%)	1	0.567

Reintervention was required in 40.0% of patients with anastomotic leakage (p = 0.064). The mean hospital stay in this group was 10.00 ± 2.83 days, compared to 8.45 ± 6.48 days in patients without leakage (p = 0.648). Although this average duration may appear short for such a severe complication, it reflects the characteristics of our cohort, which included several patients who experienced early clinical deterioration and required urgent reoperation or succumbed within the first 7 to 10 days. These cases contributed to a lower mean length of stay despite the clinical severity. Mortality occurred in 40.0% of patients with leakage versus 12.0% of those without (p = 0.183) (Table 4).

**Table 4:** Comparison of reoperation, hospital stay, and mortality between patients with and without anastomotic leakage (n = 30).

Variable	With leakage (n = 5)	Without leakage (n = 25)	p-value	F-value
Reoperation				
Yes	2 (40.0%)	-	0.064	0.023
No	3 (60.0%)	25 (100.0%)	-	-
Hospital stay (days)	10.00 ± 2.83	8.45 ± 6.48	0.648	-
Mortality				
Yes	2 (40.0%)	3 (12.0%)	0.183	0.183
No	3 (60.0%)	22 (88.0%)	-	-

Finally, serum levels of CRP and PCT were compared between patients with and without leakage. On postoperative day 5, PCT levels were higher in the leakage group (9.02 ± 15.92 ng/mL) compared to those without leakage (2.63 ± 1.52 ng/mL), with a raw p-value of 0.044. However, after correction for multiple comparisons using the Bonferroni and Holm methods, this result was no longer statistically significant (adjusted p = 0.264 for both). CRP levels followed a similar trend on day 5 (288.45 ± 223.67 mg/L in patients with leakage vs. 186.41 ± 74.83 mg/L without leakage), but the difference also did not reach significance after correction (raw p = 0.067; adjusted p = 0.402 Bonferroni, 0.335 Holm) (Table 5).

**Table 5:** Comparison of serum levels of CRP and procalcitonin between patients with and without anastomotic leakage (n = 30). P-values were adjusted using the Holm–Bonferroni method to correct for multiple comparisons. A corrected p-value < 0.05 was considered statistically significant. PCT: Procalcitonin.

Parameter (mean ± SD)	With leakage (n = 5) (ng/mL)	Without leakage (n = 25) (ng/mL)	p-value	Corrected p-value
PCT postoperative day 1	4.12 ± 2.74	4.13 ± 3.70	0.995	0.995
PCT postoperative day 3	7.70 ± 11.23	4.44 ± 4.51	0.273	0.819
PCT postoperative day 5	9.02 ± 15.92	2.63 ± 1.52	0.044	0.264
CRP postoperative day 1	167.64 ± 69.40	179.11 ± 41.94	0.621	0.995
CRP postoperative day 3	185.62 ± 99.38	170.29 ± 68.01	0.673	0.995
CRP postoperative day 5	288.45 ± 223.67	186.41 ± 74.83	0.067	0.402

## Discussion

Anastomotic leakage remains one of the most severe complications following gastrointestinal surgery, contributing substantially to patient morbidity, prolonged hospitalization, and mortality [11]. Early identification of this complication is essential for improving outcomes. In this context, the present study aimed to assess the utility of PCT and CRP as postoperative biomarkers for detecting anastomotic dehiscence in a secondary-level hospital setting.

Our findings showed that PCT levels measured on postoperative day five were higher in patients who developed leakage compared to those who did not. Although the raw p-value initially indicated statistical significance, this difference did not remain significant after applying corrections for multiple comparisons. A similar upward trend was noted in CRP levels, though this marker also failed to reach statistical significance. These results, while inconclusive, align with previous studies reporting that serial increases in inflammatory markers, particularly PCT, may reflect early infectious or inflammatory complications such as anastomotic leakage [12,13].

These observations suggest that while PCT, and to a lesser extent, CRP, could be useful in monitoring postoperative evolution, their standalone diagnostic utility remains uncertain. The loss of statistical significance after correction, coupled with the small sample size and wide variability in biomarker levels, underscores the need for caution in interpretation. Further research in larger, prospective cohorts is required to validate these preliminary findings and clarify the role of serial biomarker monitoring in clinical decision-making.

The demographic and clinical characteristics of our sample, such as mean age and comorbidity patterns, reflect the typical population undergoing intestinal anastomosis, as reported in previous studies [14,15]. Cardiovascular disease was the most prevalent comorbidity, consistent with its high burden in surgical patients of advanced age.

Neoplastic conditions and intestinal ischemia were the leading indications for surgery, aligning with the findings of Wako G et al., and Zhou C et al., who identified these as common causes for intestinal resection and anastomosis [14,15]. Elective procedures and mechanical anastomosis techniques predominated, reflecting contemporary surgical preferences due to their technical efficiency and reproducibility, as noted by Neutzling CB et al. [16].

The overall anastomotic leakage rate observed in our study was 16.6%, which falls within the range reported in the literature for colorectal procedures [17]. Among the five patients with anastomotic leakage, two cases occurred at colorectal sites, while the remaining involved ileocolic, ileorectal, and jejunoileal anastomoses. Although the sample size limits statistical inference, the predominance of leakage in colorectal locations is consistent with existing evidence suggesting increased vulnerability of low pelvic anastomoses due to factors such as reduced perfusion and surgical complexity in the confined pelvic space [18].

Although the differences in hospital stay, reoperation rates, and mortality between patients with and without leakage were not statistically significant, likely due to the limited sample size, the clinical impact remains evident. These trends are consistent with prior studies demonstrating that anastomotic dehiscence is associated with increased healthcare burden and poorer outcomes [19,20].

Although the initial unadjusted analysis showed a statistically significant difference in PCT levels on postoperative day five between patients with and without leakage, this significance was lost after applying the Holm-Bonferroni correction for multiple comparisons. This highlights the importance of interpreting isolated p-values with caution, especially in studies with small sample sizes and multiple simultaneous tests. Nevertheless, the observed trend remains clinically relevant and consistent with prior literature, supporting the need for further investigation in larger cohorts.

Limitations

This study has several limitations. First, the sample size was small (n=30), with only five confirmed cases of anastomotic leakage, which limited the statistical power to detect small or moderate associations. Second, no multivariate analysis was conducted to adjust for potential confounding variables such as age, comorbidities, or surgical technique.

Third, the reported mean hospital stay in patients with anastomotic leakage (10.00 ± 2.83 days) may appear unexpectedly short given the severity of the complication. This was due to the inclusion of patients who experienced early deterioration and either required urgent reoperation or died within the first postoperative week. These cases, while clinically severe, contributed to a lower average length of stay, which may not reflect the expected clinical course in survivors. This underscores the need for cautious interpretation of duration-related variables in small samples.

Additionally, although multiple comparisons were statistically adjusted using the Holm-Bonferroni method to reduce the risk of Type I error, this correction further limited the detection of statistically significant differences. This underscores the need for larger sample sizes in future studies to confirm these biomarker trends with adequate statistical power.

## Conclusions

Although PCT levels on postoperative day five were higher in patients with anastomotic leakage compared to those without, this difference did not remain statistically significant after correction for multiple comparisons. C-reactive protein showed a similar upward trend but also failed to reach statistical significance. Patients who developed leakage demonstrated progressive increases in these biomarkers over time, while those without leakage showed declining values.

These findings suggest a potential role for serial measurement of PCT, and possibly CRP, in the early identification of anastomotic leakage following intestinal surgery. However, due to the small sample size, wide variability in biomarker levels, and lack of statistical robustness, these preliminary results must be interpreted with caution. At present, routine clinical use of PCT as a predictive marker for leakage cannot be recommended.

Larger, prospective studies with adequate sample sizes and multivariate analyses are required to validate these findings and establish the true diagnostic performance of inflammatory biomarkers, particularly in resource-limited or secondary-care settings.
